# Microdosimetry for Targeted Alpha Therapy of Cancer

**DOI:** 10.1155/2012/153212

**Published:** 2012-09-04

**Authors:** Chen-Yu Huang, Susanna Guatelli, Bradley M. Oborn, Barry J. Allen

**Affiliations:** ^1^Centre for Experimental Radiation Oncology, St. George Clinical School, University of New South Wales, Kogarah, NSW 2217, Australia; ^2^Illawarra Cancer Care Centre, Wollongong, NSW 2522, Australia; ^3^Centre for Medical Radiation Physics, University of Wollongong, NSW 2522, Australia; ^4^Ingham Institute of Applied Medical Research, Faculty of Medicine, University of Western Sydney, Liverpool, NSW 2170, Australia

## Abstract

Targeted alpha therapy (TAT) has the advantage of delivering therapeutic doses to individual cancer cells while reducing the dose to normal tissues. TAT applications relate to hematologic malignancies and now extend to solid tumors. Results from several clinical trials have shown efficacy with limited toxicity. However, the dosimetry for the labeled alpha particle is challenging because of the heterogeneous antigen expression among cancer cells and the nature of short-range, high-LET alpha radiation. This paper demonstrates that it is inappropriate to investigate the therapeutic efficacy of TAT by macrodosimetry. The objective of this work is to review the microdosimetry of TAT as a function of the cell geometry, source-target configuration, cell sensitivity, and biological factors. A detailed knowledge of each of these parameters is required for accurate microdosimetric calculations.

## 1. Introduction

**Figure 1:**
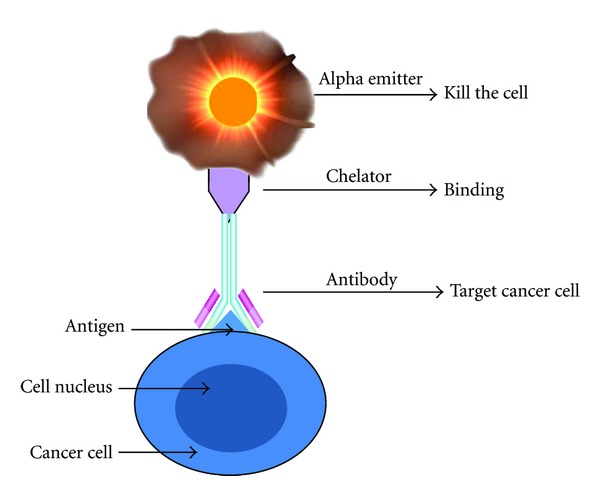
Schematic diagram of an AIC targeting a cell.

Targeted alpha therapy (TAT) can provide selective systemic radiotherapy to primary and metastatic tumors (even at a low dose rate and hypoxia region) [1]. It permits sensitive discrimination between target and normal tissue, resulting in fewer toxic side effects than most conventional chemotherapeutic drugs. Monoclonal antibodies (MAbs) that recognize tumor-associated antigens are conjugated to potent alpha emitting radionuclides to form the alpha-immunoconjugate (AIC) (Figure 1). The AIC can be administered by intra-lesional, orthotopic, or systemic injection. Targeted cancer cells are killed by the short-range alpha radiation, while sparing distant normal tissue cells, giving the minimal toxicity to normal tissue [2].

An alpha particle with energy of 4 to 9 MeV can deposit about 100 keV/*μ*m within a few cell diameters (40–90 *μ*m), causing direct DNA double-strand breaks, which lead to cancer cell apoptosis [3]. Cell survival is relatively insensitive to the cell cycle or oxygen status for alpha radiation [4]. TAT is potent enough to eradicate disseminated cancer cells or cancer stem cells that are minimally susceptible to chemo- or radio-resistance. The relative biological effect (RBE) of alpha particles is from 3 to 7 [5], which means that for the same absorbed dose, the acute biological effects of alpha particles are 3 to 7 times greater than the damage caused by external beam photons or beta radiation.

TAT is ideally suited to liquid cancers or micrometastases [6]. However the regression of metastatic melanoma lesions after systemic TAT in a phase I clinical trial for metastatic melanoma has broadened the application to solid tumors [7]. The observed tumor regression could not be ascribed to killing of all cancer cells in the tumors by TAT and led to the hypothesis that tumors could be regressed by a mechanism called tumor antivascular alpha therapy (TAVAT) [8]. Therapeutic efficacy relates to the extravasation of the AIC through porous tumor vascular walls and widened endothelial junctions into the perivascular space in the solid tumor. The AICs bind to antigenic sites on the membranes of pericytes and contiguous cancer cells around the capillary. The alpha-particle emitters are localized close to the vascular endothelial cells (ECs), which are irradiated by alpha particles and killed. Subsequent tumor capillary closure, causing depletion of oxygen and nutrition, is the likely cause of cancer cell death and tumor regression [8, 9].

## 2. Microdosimetry 

### 2.1. Microdosimetry Concept

Radiation dosimetry is the study of the physical properties of radiation energy deposition in tissue. It can be used to optimize treatments and allow comparison of different therapeutic approaches, as well as to study the basic methods of irradiation of biological matter [10]. Radiation dose in conventional external radiotherapy is a macroscopic concept. Upon the properties of the short path length alpha emissions and the spatial distribution of the radionuclide relative to the small target volumes, microdosimetry is indispensible for TAT to investigate the physical properties of radiation energy deposition in biological cells [11].

The dosimetry of TAT is distinguished from that of beta immunotherapy [12] or external beam radiotherapy in three different ways.

1. *Short path length of alpha particles*. The high energy of alpha particles is deposited in a short range [13]. Some cell nuclei receive multiple alpha particle hits, while others receive no hits. The amounts of energy deposited vary greatly from target to target, leading to a wide frequency distribution [14].
2. *Small target volume*. The alpha track length is comparable to cellular/subcellular sizes causing high LET within the small target volume. It is important to understand the differing biological effects on individual cells [15]. Given the energy delivered along an alpha-particle track and its potential cytotoxicity, the dosimetry for estimating mean absorbed dose may not always yield physically or biologically meaningful information of radiation energy deposition in biological cells. Instead, stochastic or microdosimetric methodologies may be required [16].
3. *Nonuniform distribution of radioisotopes*. The heterogeneous antigen expression and tumor uptake leads to variable spatial microdosimetric distributions of the AIC [17]. Spatial and temporal changes of the source activity in the target can also occur [4]. When the distribution of radio-labeled antibody is nonuniform, techniques of dose averaging over volumes greater in size than the individual target volumes can become inadequate predictors of the biological effect [18].

The specific energy is the most important quantity for microdosimetry as it can be used to calculate the cancer cell survival rate. Specific energy (*z* Gy) is the ratio of the energy deposited (*ε* Joule) to the mass of the target (*m* kg) (1) and has the same units as absorbed dose [19]. The mean specific energy equals the absorbed dose [15]. Although microdosimetry is concerned with the same concept of energy deposition per unit mass as dosimetry, the difference in the length of alpha particle and small size of the target volume introduces stochastic effects which are negligible in conventional dosimetry [20].

(1) $$\begin{array}{c} z=\frac{\varepsilon }{m}\;\text{Gy}. \end{array}$$

The stochastic quantity of specific energy *z* can be used to investigate biological effects [21]. The cell survival fraction (SF) is given by

(2) $$\begin{array}{c} \text{SF}\;=\;e^{-z/z_{0}}, \end{array}$$

where *z*_0_ is the absorbed dose required to reduce cell survival to 37% [22].

Many microdosimetric models have been developed since Roesch's initial work [23]. Two different microdosimetric methods can be used. Experimental measurement with high-resolution solid-state microdosimeters is one way. The high spatial resolution and the tissue-equivalence correction detectors have been applied for hadron therapy and Boron neutron capture therapy [24–26]. On the other hand, dose distributions can be calculated with analytical calculations [15] or Monte Carlo techniques based on fundamental physical principles [27]. The latter method is more practical and much less expensive [19].

### 2.2. Microdosimetry Case Study

It is inappropriate to evaluate the background dose for radioimmunotherapy, especially for TAT by conventional dosimetry. For example, in the phase I clinical trial for metastatic melanoma, patients received up to 25 mCi of ^213^Bi [7]. Assuming that all the activity injected in patient remains in the blood, 25 mCi corresponds to 3.65 × 10^12^ ^213^Bi atoms in the macrodosimetry point of view. Taking the average energy of 8.32 MeV, the total energy lost would be 4.8 Joules. The absorbed dose (*z*) received by blood (5 L) would be calculated by

(3) $$\begin{array}{c} z=\frac{\varepsilon }{m}=\frac{4.8\;\text{J}}{5\;\text{kg}}\approx 1\;\text{Gy}. \end{array}$$

The above dosimetry would indicate that the blood system would receive an absorbed dose of ~1 Gy from alpha particle irradiation. The risk for unwanted radiation exposures of normal tissues would be too high.

However, by using Geant4 Monte Carlo microdosimetry calculation, the actual dose to endothelial cells is ~2 cGy and to lymphocyte is ~10 cGy [28], being 2% and 10% of the macroscopic absorbed dose, respectively, and is too low to post any serious damage. In other words, unless alphas actually hit their target, their energy is lostto the medium and has no effect on normal tissue. 

Memorial Sloan Kettering found that the maximum tolerance dose was in excess of 1 mCi/kg for bone marrow toxicity [29]. In the melanoma clinical trial [4], 25 mCi converts to ~0.3 mCi/kg and is well below that value.Thus, the clinical trial result is consistent with the Monte Carlo calculations. The entirely different result from two dosimetry methods shows it is essential to use microdosimetry method for TAT.

## 3. Factors Affecting TAT Microdosimetry Result

### 3.1. The Choice of the Target

Microdosimetry depends on the choice of target, for example, the entire cell, cell nucleus, or DNA. This is because the size of the target will affect both the energy deposition and the mass which determines the specific energy.

As has been substantiated by* in vitro* and *in vivo* experiments, the radiosensitive sites are associated with DNA in the cell nucleus. Microdosimetry research target has fluctuated between DNA and cell nucleus. For alpha particles with a range of a few cell diameters, the cell nucleus is an appropriate choice for the target considering the genome is assumed to be randomly distributed throughout the cell nucleus, and its specific location is not well known [20]. However, under the circumstances that if the particle (e.g., Auger electrons) range is a few *μ*m and the source decays within 1 or 2 nm of the DNA molecule, radiation dose to the cell nucleus may be inadequate to predict radiation toxicity, and determination of the energy deposition to the DNA molecule may be necessary [30].

### 3.2. Target and Source Configuration

The TAT microdosimetry dose is highly sensitive to experimental factors such as the nucleus size and source distribution, kinetics of the AIC, and subcellular distribution of the radionuclide. 

Because of the short range of alpha particles, even small changes in the thickness or diameter of the cell nucleus can influence the dose distribution. Simplified spheroid models with different cell and nucleus radii are used to model cells [4, 15, 31]. Recently some more realistic models with geometric parameters taken from monolayer or suspended cells measurement were built [32–35]. 

The position of the source relative to the target cell nucleus is another major factor in determining the hit probability, specific energy, and ultimately the efficacy of TAT [36]. For a spherical single-cell model, the specific energy from activity internalized in the cell nucleus, in the cytoplasm, on the cell membrane, or in the medium can differ as much as 150 times [37]. Rapidly internalized antibodies or radioisotopes are superior because of markedly greater intracellular retention and higher probability of hit. However, caution is needed as some animal studies indicate that the retention of ^111^In and ^90^Y is prolonged in normal organs such as bone, liver, and kidney as well [38].

### 3.3. Cell Sensitivity

The *z*_0_ value is highly sensitive to experimental factors such as the distribution of DNA within the nucleus (i.e., the phase of the cell cycle) and the number and spatial distribution of the alpha particle sources relative to the target cell nucleus [39]. It is also expected that the *in vitro* cell sensitivity will vary between different cells within a given tumor.  Table 1 shows a survey of TAT *in vitro* experiments, which illustrated that *z*_0_ values for cancer cell lines exposed to alpha radiation can vary as much as 6 times, indicating that for a given specific energy, the biological responses can also vary 6 times or more (SF in (2)). 

### 3.4. Radioisotopes

The clinical application of TAT is focused on alpha particle emitters of ^211^At, ^213^Bi, ^223^Ra, and ^225^Ac [40–42]. The physical properties of these radionuclides, including half-life, mean particle energy, maximum particle energy, and average range in tissue, affect the therapeutic result and are listed in Table 2 [43, 44].

For an AIC with a long residence time in a tumor, a radionuclide with a long half-life will deliver more decays than one with a short half-life (*t*_1/2_) for the same initial radioactivity [44]. The number of radionuclides (*N*) to produce activity (*A*) is

(4) $$\begin{array}{c} N=\frac{A}{\lambda }=\frac{A}{\left(0.693/t_{1/2}\right)}. \end{array}$$

For TAT aimed at destroying all cancer cells in the tumor, deep penetration and uniform distribution of the AIC would be crucial. Thus, the longer half-life radioisotope would be a better choice. However, if the aim is to destroy tumor capillaries, poor AIC diffusion away from the capillaries and shorter half-life would be an advantage [19].

**Figure 2:**
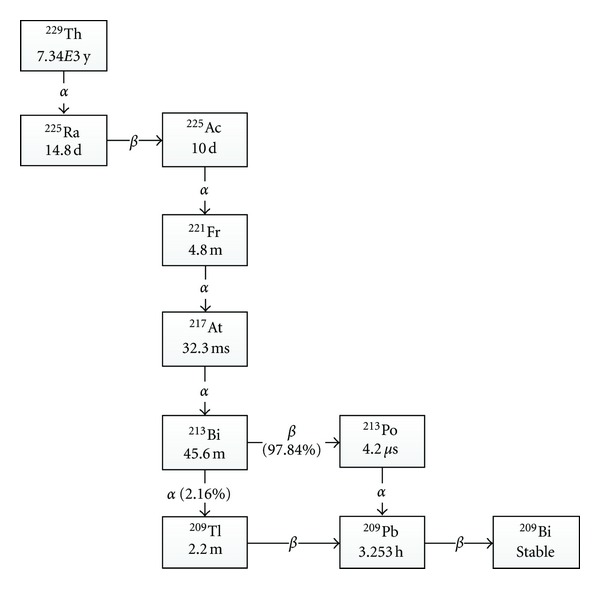
The decay chain of ^225^Ac and ^213^Bi.

The longer half-life of ^225^Ac and the 4 alpha particle emissions (Figure 2) gives greater toxicity and can prolong survival in the mouse xenograft models for several cancers [45]. However, the drawback is that the binding energy of the chelation is not strong enough to withstand the alpha particle recoil energy of the Actinium ion (between 100 and 200 keV). Daughters of ^225^Ac will lose tumor selectivity and could diffuse away, causing cell damage in normal tissue [16, 46].

### 3.5. Biological Factors

There are great complexities of the mammalian cell, the nucleus and its internal structures and pathways, types of DNA damage, and cellular repair. For cancer-cell cluster or solid-tumor modeling, a precise kinetic description of AICs diffusing through cells and saturating antigenic sites is needed for microdosimetry. The number of AICs in the solid tumor depends critically on the capillary permeability and the number of antigens expressed on cells that can vary 10-fold and more. As such, the calculations rest on realistic assumptions but results, in spite of the quantitative nature of the Monte Carlo calculation, are qualitative only.

## 4. Conclusion

Targeted Alpha Therapy uses radio-isotopes that emit alpha radiation to kill targeted cancer cells. It is most effective in the elimination of single-cancer cells and micrometastases before the tumors grow to become clinically evident. The application has been extended to the treatment of solid tumors by tumor antivascular alpha therapy. Because the short range of alpha particles is comparable to the size of the biological target and the variable distribution of alpha emitters, stochastic processes apply, and Monte Carlo calculations of microdosimetry are indispensible in the investigation of biological response mechanisms. 

## Figures and Tables

**Table 1:** Experimental values of *z*_0_ for *in* *vitro* exposure to alpha radiation.

Cell line	AIC	*z*_0_ (Gy)	Reference
MCF7	^ 225^Ac-Herceptin	0.27	[47]
BT	^ 225^Ac-Herceptin	0.37	[47]
MDA	^ 225^Ac-Herceptin	0.53	[47]
Line 1	^ 213^Bi-13A	1.4	[36]
EMT-6	^ 213^Bi-13A	1.7	[36]

**Table 2:** Physical properties of alpha-particle emitters.

Radionuclide	*Z*	Half-life	Mean particle energy* (MeV)	Maximum energy (MeV)	Average range (*μ*m)	<LET> (keV/*μ*m)
^ 211^At	85	7.2 h	6.79	7.45	60	71
^ 213^Bi	83	45.6 min	8.32	8.38	84	61
^ 223^Ra	88	11.43 d	5.64	7.59	45	81
^ 225^Ac	89	10.0 d	6.83	8.38	61	71

*weighted average of emissions.
